# Universal health coverage and chronic kidney disease in India

**DOI:** 10.2471/BLT.18.208207

**Published:** 2018-07-01

**Authors:** Beverley M Essue, Vivekanand Jha, Oommen John, John Knight, Stephen Jan

**Affiliations:** a Menzies Centre for Health Policy, The University of Sydney, Sydney NSW 2006 A, Australia.; b The George Institute for Global Health, New Delhi, India.; c The George Institute for Global Health, Sydney, Australia.

Kidney diseases are associated with an estimated 188 million cases of catastrophic health expenditure in low- and middle-income countries.^1^ The scale of the burden associated with this condition in these countries demands action. Kidney diseases disproportionally affect disadvantaged populations^2^ and reduce the number of productive years of life.^3^ Furthermore, the prospect of financial burden discourages many patients from undergoing treatment, thereby leading to preventable morbidity and death.

The impact of kidney diseases has been quantified in India, in a cohort of 119 working-age dialysis patients, most of whom lacked health insurance.^4^ In this cohort, 35/119 (29%) patients died and 16/119 (13%) discontinued dialysis within 12 months. Despite receiving highly subsidized treatment, dialysis patients receiving care in these two sites in northern India still faced high medical out-of-pocket costs: 87.1% of patients in public hospitals were spending over 100% of their monthly income on dialysis compared to 78.9% of patients in private care.^4^ This expenditure excluded non-medical costs, which can also be substantial.^5^

As part of its agenda to achieve universal health coverage (UHC) by 2022, the Indian government has committed to establishing at least one eight-station dialysis unit in each of its 688 districts, and is offering free haemodialysis to people living below the poverty threshold.^6^ The government’s ability to meet this commitment will depend not only on increasing its fiscal capacity, but also on the implementation of frugal innovations (such as low-cost dialysis machines^7^ and greater use of non-physician health workers), enhanced early screening interventions^8^ and better access to home-based peritoneal dialysis. Better access to peritoneal dialysis would potentially mitigate the substantial non-medical costs associated with travel and lost productivity to attend haemodialysis units.^5^^,^^6^

While financing reforms to implement UHC are critical to enhancing financial protection of patients with chronic kidney disease, these reforms are not enough. Dialysis and transplantation are highly unaffordable in most low- and middle-income countries, particularly for vulnerable groups.^6^ Comprehensive health benefit packages must prioritize early screening and treatment of risk factors such as diabetes and hypertension, access to essential medicines and the implementation of public health interventions to prevent disease progression.^8^ Targeted support programmes are also needed as part of a comprehensive strategy to strengthen financial protection for chronic kidney disease patients. One such programme has been introduced by the Government of Andhra Pradesh and provides grants of 2500 Indian rupees (35 United States dollars) to eligible chronic kidney disease patients to offset out-of-pocket costs,^9^ providing a potentially useful model for other Indian states.

Coverage for kidney disease care represents perhaps the greatest challenge in achieving UHC in India and most other low- and middle-income countries.^10^ This is due to the high cost of existing treatments, high disease burden and constrained financial resources. While reforms are needed to better finance health-care services, including dialysis, much can be done to address the economic burden through greater emphasis on prevention and the development of low-cost treatments.^7^ Most individuals who currently have chronic kidney disease in India face catastrophic health expenditure.^1^ Achieving UHC will enable timely access to care and prevent thousands of households from falling into poverty due to kidney disease each year.^1^
